# Hemodynamic and Neuropathological Analysis in Rats with Aluminum Trichloride-Induced Alzheimer's Disease

**DOI:** 10.1371/journal.pone.0082561

**Published:** 2013-12-20

**Authors:** Szu-Ming Chen, Chi-Chen Fan, Ming-Shiuan Chiue, Chi Chou, Jyh-Horng Chen, Ruey-Shyang Hseu

**Affiliations:** 1 Department of Biochemical Science and Technology, College of Life Science, National Taiwan University, Taipei, Taiwan; 2 Department of Medical Laboratory Science and Biotechnology, Yuanpei University, Hsinchu, Taiwan; 3 Interdisciplinary MRI/MRS Lab, Department of Electrical Engineering and Molecular Imaging Center, National Taiwan University, Taipei, Taiwan; 4 Graduate Institute of Biomedical Engineering, National Taiwan University, Taipei, Taiwan; 5 Department of Physiology, Mackay Memorial Hospital, Taipei, Taiwan; University G. D'Annunzio, Italy

## Abstract

**Background and Aims:**

Hemodynamic normality is crucial to maintaining the integrity of cerebral vessels and, therefore, preserving the cognitive functions of Alzheimer's disease patients. This study investigates the implications of the hemodynamic changes and the neuropathological diversifications of AlCl_3_-induced AD.

**Methods:**

The experimental animals were 8- to 12-wk-old male Wistar rats. The rats were randomly divided into 2 groups: a control group and a (+)control group. Food intake, water intake, and weight changes were recorded daily for 22 wk. Synchronously, the regional cerebral blood flow (rCBF) of the rats with AlCl_3_-induced AD were measured using magnetic resonance imaging (MRI). The hemorheological parameters were analyzed using a computerized auto-rotational rheometer. The brain tissue of the subjects was analyzed using immunohistological chemical (IHC) staining to determine the beta-amyloid (Aβ) levels.

**Results:**

The results of hemodynamic analysis revealed that the whole blood viscosity (WBV), fibrinogen, plasma viscosity and RBC aggregation index (RAI) in (+)control were significantly higher than that of control group, while erythrocyte electrophoresis (EI) of whole blood in (+)control were significantly lower than that of control group. The results of acetylcholinesterase-RBC (AChE-RBC)in the (+)control group was significantly higher than that of the control group. The results also show that the reduction of rCBF in rats with AlCl_3_-induced AD was approximately 50% to 60% that of normal rats. IHC stain results show that significantly more Aβ plaques accumulated in the hippocampus and cortex of the (+)control than in the control group.

**Conclusion:**

The results accentuate the importance of hemorheology and reinforce the specific association between hemodynamic and neuropathological changes in rats with AlCl_3_-induced AD. Hemorheological parameters, such as WBV and fibrinogen, and AChE-RBC were ultimately proven to be useful biomarkers of the severity and progression of AD patients. In addition, the parameters can be substituted for invasive inspection in therapeutic intervention.

## Introduction

Alzheimer's disease (AD) is the most common form of dementia among the elderly population that causes a gradual decline in cognitive abilities. According to the “amyloid cascade,” amyloid plaques are formed through the abnormal aggregation of beta amyloids (Aβs) [1], which are deposited at the extracellular spaces of the brain and the walls of the cerebral blood vessels. Amyloid plaques increase levels of oxidative stress and neuroinflammation, and markedly reduce acetylcholine levels. They are crucial histological characteristics of the pathology of AD. The abnormal aggregation of Aβ is a primary cause of the progressive changes of AD.

In the past few years, AD has been recognized as a degenerative disease of the central nervous system. However, recent evidence has shown the disturbance of the cerebrovascular and systemic vascular systems (vasculopathy) in AD patients. AD patients with a history of cerebrovascular disease are likely to have the disease develop rapidly. Evidence from brain imaging studies using cerebral computed tomography perfusion imaging (CTPI) [2], single-photon emission computed tomography (SPECT) [3], and MRI [4] has shown reduced regional cerebral blood flow (rCBF) in AD groups. Studies have shown that AD patients exhibit remarkable anomalies in hemodynamic parameters [5], [6], [7] and several substantial hemorheological changes caused by the accumulations of Aβ [8], [9], which may initiate the changes on the cerebrovascular structure that cause the microvascular plasma layer of the brain to fail to deliver glucose, oxygen, amino acids, electrolytes, and other nutrients through the blood-brain barrier. Because of the low metabolism of glucose and the lack of oxygen delivery to the neurons of the brain, the neurons cannot receive the required amount of energy, leading to the death of the neuron and the deterioration of cognitive functions [6], [10]. The cerebral microvasculature is a crucial target for the effects of hypoxia in the AD brain [11]. Hemorheological detections provide the most direct evidence for systemic vascular disturbances in AD patients. The hemorheological behavior of AD patients has also been reported. Significant differences exist in all hemorheological indices except hematocrit HCT between an AD group and a control group [12], [13]. These hemorheological changes are some of the major vascular risk factors. The treatment of vascular risk factors is associated with a slow decline in Mini-Mental State Examination score in AD patients [14].

Aluminum (Al) is considered part of the etiology of AD [15]–[17]. An excess amount of Al causes amyloid neurotoxicity according to records of clinical observation and animal experiments [18]. Past animal studies have shown that Al-induced damages to the central nervous system include neuropathological, neurochemical, neurophysiological, and neurobehavioral changes. Among the changes, the most notable are poor learning and behavioral functions, which involve a change in acetylcholinesterase activity that deteriorates the learning ability of rats [19]. Excessive intake of Al may cause the deposition of amyloids in the central nerve cells, excessive APP expression, and learning and memory disorders in rats [20], [21]. The neurotoxic effects of Al directly affect the function of glial cells (astrocytes) [22]. However, the exact mechanism of Al-induced dysfunction of the cerebral microcirculature is unclear. Therefore, we orally administered AlCl_3_ to rats [23], [24] to explore the hemodynamic and neuropathological changes of AD.

In this study, we used the difference-learning experimental model Morris water maze test to assess the learning and memory ability of rats with AlCl_3_-induced AD. Immunohistochemical (IHC) methods were applied to observe the Aβ plaque levels, detect the activity of AChE-RBC- and AChE-hippocampus, and quantitate the content of cerebral neurochemicals by using proton magnetic resonance spectroscopy (1H-MRS). The 1H-MRS allows major metabolites to be measured noninvasively in defined regions of the living brain and can be used to detect biochemical abnormalities where conventional structural imaging shows normalities [25]. Therefore, we measured hemorheological parameters by using an auto-rotational viscometer with a different shear rate and detected rCBF by using arterial spin labeling MRI (ASL-MRI). Recently, ASL-MRI was used to measure hemodynamic parameters in patients with AD [26]. We propose the hypothesis that numerous Aβ plaques increase erythrocyte aggregation and whole blood viscosity, which reduces rCBF. This impairment of rCBF adversely affects the activity of neurons. Large accumulation of Aβ plaques can increase whole blood viscosity and the likelihood that cerebral degeneration will spread. In other words, we propose an Aβ-correlated hemodynamic cascade hypothesis to elucidate the vital mechanisms of hemorheology and neurovascular pathology in AD.

## Materials and Methods

### Animal Model Preparation and Experimental Design

Twenty male Wistar rats weighing 220–260 g and aged 8–12 wk were procured from BioLasco Taiwan Co., Ltd. The Morris water maze test was used to exclude rats that performed abnormally. The 20 qualified rats were randomly divided into 2 groups: control (n = 10) and (+)control (n = 10). Animal handling and experimental procedures were approved by the Institutional Animal Care and Use Committee (IACUC Approval No: 20120307).

The rats were administered daily aluminum chloride 500 mg/kg, i.g, for one month, and were subsequently fed with AlCl3 solution (1600 ppm in distilled water) for up to 5 mo.

The AlCl_3_-induced brain dysfunction models were established 5 mo after the oral administration of Al. The Morris water maze test was used again to evaluate the learning and memory functions of the rats. ASL-MRI was used to calculate cerebral blood flow, MRS was used to detect cerebral metabolism, and magnetic resonance angiography (MRA) was used to detect changes in brain vessel morphology. Finally, the rats were euthanized. Simultaneously, blood from jugular and abdominal veins was collected to measure hematological, hemorheological, and biochemical parameters, and brain tissues were placed in formalin to observe the Aβ plaque levels by using IHC staining method.

### Morris Water Maze Task

The water maze compriseda large black circular pool (180 cm in diameter, 75 cm in height) constructed using waterproof canvas, and a clear acrylic platform (20 cm in diameter, 47 cm in height) was placed inside the pool. The pool was filled to a height of 49 cm with water at approximately 23°C, and the surface of the platform was 2.0 cm below the surface of the water. The circular pool was divided into 4 quadrants (I, II, III, and IV), the platform was hidden in the middle of the 4th quadrant, and a camera connected to a computer to record the animal motions was positioned above the center of the pool. The rats were trained twice a day for 5 d, then once every month after being treated with AlCl3 solution. The escape latency and searching distance were used to evaluate learning and memory functions, it was named the “reference memory task”. On Day 1, the adaptation period, the rats were placed in the water pool with no platform that could be used to escape and allowed to swim freely for 2 min. On Days 2 to 4, the water maze reference memory task was performed. First, the rats were placed in the pool 4 consecutive times and allowed to swim freely. The rats were then placed in the pool in one of the 4 quadrants (by 2→1→3→4 quadrant) and were required to find the platform to escape. The time taken to escape from the water (escape latency) and the path crossed in the water (searching distance) were measured and analyzed using an automated tracking system—Ethovision® XT,Version 8.0,Noldus system (Neuroscience Inc., Tokyo, Japan).The maximal escape latency time was specified as 120 s. If a rat was incapable of finding the platform within 120 s, the rat was guided to the platform and remained there for 30 s. When the trial was completed, the rat were removed from the water, towel and heater dried, and then returned to the original squirrel cages. On Day 5, the “water mazespatialprobe trial” was performed. The rats were placed in the water pool with no platform that could be used to escape and allowed to swim for 120. The swimming time and search distance of each rat in each quadrant was recorded. If the rats exhibited normal memory ability, they lingered in the platform quadrant (4th quadrant).

### MRI Measurements for Regional Cerebral Blood Flow, Angiography, and Cerebral Neurochemicals

The rats were placed in a cradle equipped with a stereotaxic holder, and a pressure probe was employed to monitor respiration. A whole-brain imaging protocol involving T2-weighted imaging (T2-WI), MRS, time-of-flight (TOF) MRA, and an arterial spin labeling cerebral blood flow (ASL-CBF) test was adapted from a previous study [7]. MRI was performed using the BioSpec70/30(7T) system (Bruker, Ettlingen, Germany) with a birdcage head coil (inner diameter = 72 mm) for RF transmission and a phase array surface coil for reception in the MRS and TOF-MRA tests. A quadrature birdcage volume coil (inner diameter = 72 mm) was used for RF transmission and reception simultaneously in the ASL-CBF test. The T2-WI sequences were acquired using a TurboRARE-T2 sequence with a matrix = 384×384, an FOV = 2.5×2.5 cm, 21 slices (slice thickness = 1 mm), TE/TR = 33/2500 ms, and 4 averages. The MRS data were acquired using a PRESS-1H sequence with TE/TR = 50/2000 ms, VOI = 2.3×2.3×2.3 mm (focus on the striatum area), a band width (BW) = 50 000 Hz, and a water suppression = VAPOR. TOF MRA was performed using a FLASH-3D-TOF sequence with a matrix = 256×256, FOV = 2.5×2.5×3.5 cm, TE/TR = 2.5/15 ms, a flip angle = 20°, and NEX = 1. ASL was performed using a flow-sensitive alternating inversion-recovery echo planar imaging (FAIR-EPI) sequence with a matrix of 128×128, FOV = 2.5×2.5 cm, an inversion recovery time (TIR) = 100 to 6000, 60 TIR values, recovery time = 10 000 ms, TE/TR = 25/18 000 ms. All image analyses were performed using Paravision software (Bruker, Ettlingen, Germany) on an MRI console. The Paravision 5.1 software platform (Bruker, Ettlingen, Germany) was used in the analysis of MRS, and processing was conducted using the TOPSPIN interface. Paravision 5.1 software was used in the source images and maximal intensity projection for MRA. The ASL images were analyzed using ASL Perfusion Processing program macro from Paravision software at a 7T blood T1 value of 2200 ms [16]. CBF (in mL/[min 6100 g]) was derived from the non-selective and selective T1 maps according to a CBF = **λ**. T1 non-selective/T1 blood (1/T1 selective-1/T1 non-selective), where **λ** is the blood-brain partition coefficient (i.e., the ratio between water concentration per gram of brain tissue and per millimeter of blood), which is estimated to be 90 mL/100 g.

### Preparation of Blood and Brain Samples

After an MRI was performed, all rats were anaesthetized with sodium pentobarbital, and the blood samples were collected for biochemical, immunological, hematological, and hemorheological analyses. The brain tissues were placed in a 10% formalin-fixed buffer at 4°C. The cerebral cortex and hippocampus were separated from the whole brain and put into a 0.9% sodium chloride solution at 4°C. The samples were then homogenized with a homogenizer centrifuged at 10 000 *g* for 10 min, and the supernatant was used for oxidative stress and neurochemical assays.

### Hematological and Hemorheological Measurements

A complete blood count (CBC), which measures the number of blood cells (red blood cells, white blood cells, and platelets), the total amount of hemoglobin (Hb), the fraction of blood composed of red blood cells (hematocrit; Hct), the average red blood cell size (MCV), Hb amount per red blood cell (MCH), and the amount of hemoglobin relative to the size of the cell (Hb concentration) per red blood cell (MCHC), was obtained using an automatic cell counter (Coulter LH750, Beckmen).

### Hemorheological Variables

Whole blood viscosity (WBV,ηb), plasma viscosity (ηp), the erythroctye aggregation index (AI), and the erythroctye rigidity index (RI) were measured using a computerized auto-rotational rheometer (HRD). The WBV measurements were implemented according to the protocols outlined by standardized hemorheological methods [27]. The sequential WBV values at different shear rates (high shear rate = 120 s^−^, medium shear rate = 70 s^−^, and low shear rate = 30 s^−^) were obtained for comparison between rats with AlCl_3_-induced AD and the controls, and was conducted using a computer-controlled testing program. The internal viscosity of erythrocyte Tk was calculated using Dintenfass's equation: Tk = [(η0.4–1)η0.4]/Hc. Plasma viscosity was detected at a high shear rate of 120 s, and the plasma fibrinogen was detected using the thrombin clot technique. The oxygen transport efficiency (OTE) of whole blood was calculated using the formula OTE = (Hct/ηb)×100% at a fixed shear rate [28], [29].

### Biochemical and Immunological Analysis

The glucose, triglyceride, cholesterol, HDL-C, total protein, albumin, AST, ALT, Alk-P, and folic acid of serum were analyzed using a modular automatic biochemical analyzer (P800, Roche). The plasma homocysteine was analyzed using an automatic biochemical analyzer (7150, Hitachi). The AChE activity of RBCs was determined using an enzyme substrate method. The AChE activity of the hippocampus and cortex were determined using a choline oxidase method from a commercial kit (Amplex® Red Acetylcholinesterase Assay Kit, Invitrogen, USA).

### Glutathione Peroxidase, Superoxide Dismutase, Catalase, and MDA Assays

The mentioned supernatant from the hippocampus and cortex homogenates was used for neurochemical assay. The assay of glutathione peroxidase activity was performed by using the method of cumene hydroperoxide. The assay of SOD activity was conducted by using the method of xanthine oxidase. The assay of catalase activity was executed according to the method of hydrogen peroxide. The MDA content was analyzed by using the method of thiobarbituric acid (TBA) colorimetric analysis. All of these methods were performed according to the instructions included in the commercial assay kits (BioVision, USA).

### IHC Stain and Signal Analysis of Aβ40 in the Hippocampus and Cortex

The brain tissue specimens of rats were fixed in a 10% formalin solution and embedded in paraffin sections with a thickness of 4 µm, which were prepared from paraffin blocks and collected sequentially without interrupting the wells. Sections from regions containing the hippocampal and cortex tissue were processed for Aβ40 IHC staining. The analysis was conducted using an automatic staining machine (BenchMark XT, Ventana Medical Systems, Tucson, AZ, USA) and the iVIEW 3, 3-diaminobenzidine (DAB) detection Kit (Ventana Medical Systems). After the brain tissue sections (4 µm) on the slides were deparaffinized and hydrated, the slides were treated with an iVIEW inhibitor at 37°C for 4 min to inactivate the endogenous peroxidase activity. The slides were then incubated with an Aβ peptide N-terminal antibody (clone NT 3F5, mouse monoclonal, Mybiosource) and diluted in a 1∶150 blocking solution at 37°C for 16 min. After rinsing with PBS, the slides were treated with iVIEW biotin-conjugated IgG in a blocking solution for 8 min at room temperature. The slides were rinsed again and subsequently incubated with iVIEW streptavidin-conjugated HRP in the blocking solution for 8 min at room temperature. The Aβ peptide signals were developed in iVIEW DAB and hydrogen peroxide for 8 min at 37°C. The slides were finally incubated with iVIEW copper for 4 min to enhance the signal intensity, and were counterstained with hematoxylin (Vector Laboratories, Burlingame, CA). The Aβ40 accumulation in the hippocampus was monitored using a snatch microscope examination.

### Statistical Analysis

All data were processed using a statistical software package, SPSS17 (Chicago, IL, USA). The results were expressed as the mean **±**standard deviation (mean ± SD), and the Wilcoxin rank-sum test (*α* = 0.05) was used for the comparison of the means. A *P* value less than 0.05 was considered statistically significant.

## Results

### Comparing the Environmental Conditions of the Animals and Water Maze Results

The body weight increased significantly after 5 mo in each group, but no significant differences were found between the control and (+)control groups. The water intake (mL/d) and food intake (g/d) of rats in the control and (+)control groups also showed no significant differences.

**Figure 1:**
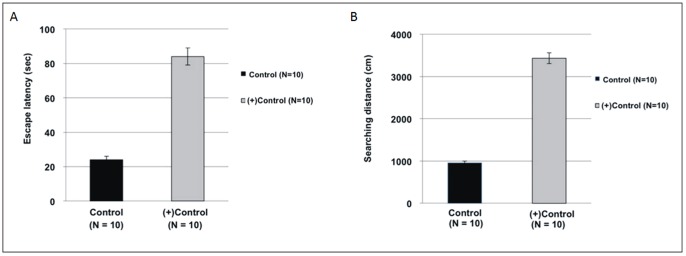
Reference memory task of water maze results. The escape latency (**A**) and searching distance (**B**) were used to evaluate the learning and memory functions of the rats. The results showed that the (+)control group exhibited a longer escape latency and searching distance than did the control group (*P*<0.05).

**Figure 2:**
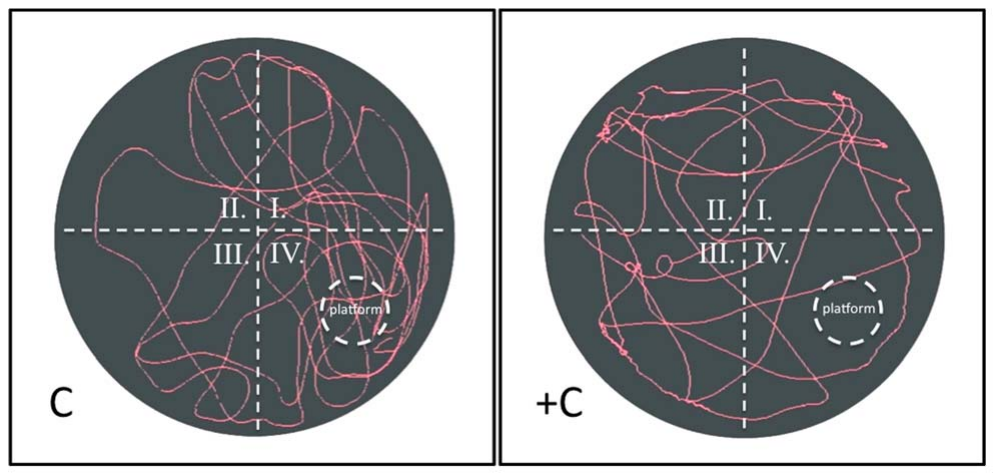
Water Maze Spatial Probe Trial. The swim pathway was instrumental in determining the learning and memory capacities of the rats. The results show that the (+)control group searched the target quadrant in a directionless manner and swam around the entire pool. Conversely, the control group swam directly to the target quadrant and lingered for a long time because of their superior memory and learning abilities.

Regarding the water maze results, the (+)control rats always exhibited a longer escape latency and searching distance than control rats (Figure 1). The spatial probe trial was conducted following the reference memory trial. We found a significant difference between the control and (+)control groups in the mean time spent in the target quadrant and the contralateral quadrant. The (+)control rats spent less time searching the target quadrant than did the control rats (*P*<0.05). The swim pathway was instrumental in determining the learning and memory capacities of the rats in the spatial probe trial. Figure 2 shows that rats in the (+)control group searched the target quadrant in a directionless manner and swam around the entire pool.

### Results of Blood Biochemical Analysis

The results of blood biochemistry analysis reveal that the concentrations of fibrinogen, AChE-RBC, AChE-hippocampus and AChE-cortex in the (+)control group were significantly higher than that in the control group. No significant differences were found in glucose, triglyceride, cholesterol, HDL-C, total protein, albumin, AST, Alk-P, homocysteine, and folic acid between the control and (+)control groups (Table 1).

**Table 1:** The results of the biochemical analysis of blood in the control and (+)control groups.

Parameters	Control (n = 10)	(+) Control (n = 10)	P Value
Glu (mg/dl)	115.8±17.3	113.7±14.8	N/S
TG (mg/dl)	70.8±12.8	69.9±13.9	N/S
Chol (mg/dl)	83.1±15.1	84.2±12.4	N/S
HDL-C (mg/dl)	56.7±6.5	54.8±7.3	N/S
TP (g/dL)	6.1±0.5	6.2±0.3	N/S
ALB (g/dL)	3.8±0.3	3.9±0.2	N/S
AST (U/L)	133.0±16.9	135.4±17.3	N/S
ALT (U/L)	44.0±9.9	45.8±8.8	N/S
Alk-P	47.9±11.2	49.3±13.9	N/S
Fibrinogen (mg/dl)	184.0±6.9	197.8±7.3	<0.05
Hcy (µmol/L)	19.4±1.4	20.3±1.7	N/S
Folic acid (ng/ml)	23.5±2.1	23.6±2.2	N/S
AChE-RBC (mole/min.L)	2.36±0.17	4.91±0.15	<0.01
AChE-hippocampus (mU/ml/mg)	55.51±6.24	91.77±9.63	<0.01
AChE-cortex (mU/ml/mg)	15.16±0.84	19.38±0.90	<0.01

Glu: glucose; TG: triglyceride; Chol: cholesterol; HDL-C: high-density lipoprotein-cholesterol; TP: total protein; ALB: albumin; AST: aspartate aminotransferase; ALT: alanine aminotransferase; Alk-P: alkaline phosphatase; Hcy: homocysteineAChE: acetylcholinesterase.

Data are presented as mean ± SD.

### Oxidative Stress in the Rats with AlCl_3_-induced AD

This study examined glutathione peroxidase, superoxide dismutase, catalase activities, and MDA content. Table 2 shows that the activities of glutathione peroxidase, superoxide dismutase, and catalase were reduced in the hippocampus and the supernatant of the cerebral cortex of the rats with AlCl_3_-induced AD (*P*<0.01), and that the MDA content increased in the supernatant of the hippocampus and cerebral cortex and the erythrocytes of the jugular blood of rats with AlCl_3_-induced AD.

**Table 2:** The activities of glutathione peroxidase, superoxide dismutase, and catalase reduced in the supernatant of the hippocampus and cerebral cortex of rats with AlCl_3_-induced AD (*P*<0.05), and the MDA content increased in the supernatant of the hippocampus and cerebral cortex and the erythrocytes of the jugular blood of rats with AlCl_3_-induced AD.

Parameters	Control group (n = 10)	(+)Control group (n = 10)	P Value
SOD activity in hippo (Inhibition rate/mg)	18.05%±0.84%	10.43%±0.99%	<0.01
SOD activity in cortex (Inhibition rate/mg)	2.84%±0.25%	1.36%±0.19%	<0.01
Catalase Activity in hippo (mU/ml/mg)	0.189±0.01	0.087±0.009	<0.01
Catalase Activity in cortex (mU/ml/mg)	0.050±0.006	0.026±0.004	<0.01
GPx activity in hippo (mU/ml/mg)	14.52±0.10	8.00±0.67	<0.01
GPx activity in cortex (mU/ml/mg)	1.18±0.01	0.03±0.004	<0.01
MDA in hippo (nmol/mg)	0.028±0.005	0.117±0.010	<0.01
MDA in cortex (nmol/mg)	0.044±0.006	0.126±0.010	<0.01
MDA in RBCs of jugular blood (nmol/mg)	1.06±0.04	3.87±0.12	<0.01

Activities of superoxide dismutase (SOD), catalase, glutathione peroxidase (GPx), and malondialdehyde (MDA) in the control and (+) control groups.

Data are presented as mean ± SD.

### MRI Acquisition and Data Analysis

**Figure 3:**
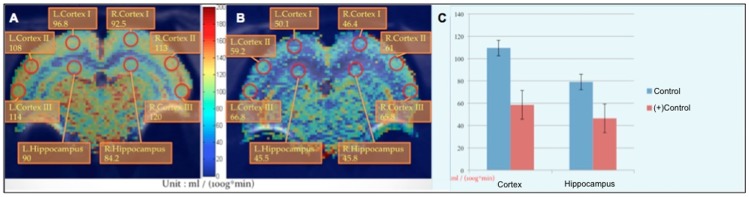
rCBF values of the hippocampus and cortex region. Images of control rats (**A**) and rats with AlCl_3_-induced AD (**B**) show that the rCBF decreased by approximately 40% to 50% in rats with AlCl_3_-induced AD compared with control rats (**C**).

**Figure 4:**
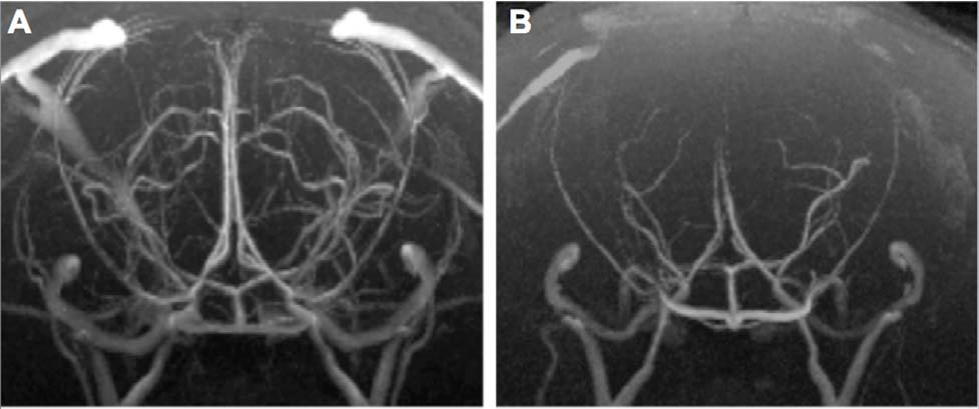
MRI angiography (TOF-MRA) results of the 2 groups of rats. Compared with control rats (**A**), most vessels around the hippocampus and cortex cannot be observed in rats with AlCl_3_-induced AD (**B**).

**Figure 5:**
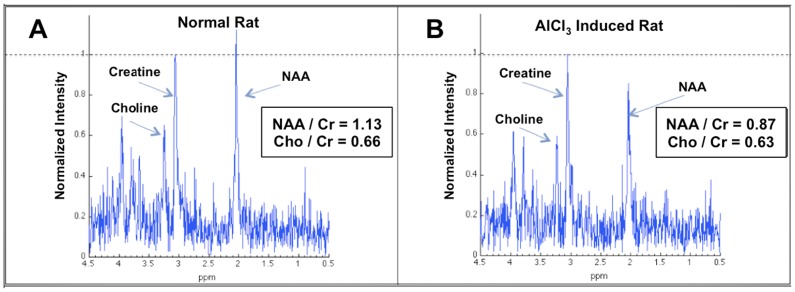
Comparison of the NAA content of the 2 groups. The NAA concentration in rats with AlCl_3_-induced AD (**B**) was significantly 10%–20% lower than in control rats (**A**).

In this study, we compared the rCBF images, TOF-MRA images, and the metabolism of the brain according to MRS. The rCBF and NAA value of rats with AlCl_3_-induced AD were quantified. The rCBF values in the cortex and hippocampus decreased by 40%–50% in rats with AlCl_3_-induced AD (Figure 3). The brain blood vessel signal of rats with AlCl_3_-induced AD was too low to be detected using TOF-MRA; the brain vessel of control rats was considerably high density whereas the (+)control group exhibited a lower density in the image (Figure 4). Furthermore, when detecting the metabolism of the rat brain by using MR spectroscopy, we used the ratio of NAA and creatine (Cr) to determine the NAA concentration (Figure 5). The NAA of rats with AlCl_3_-induced AD was approximately 30% lower than that of control rats.

### Hemorheological Analysis in Rats with AlCl_3_-induced AD

**Figure 6:**
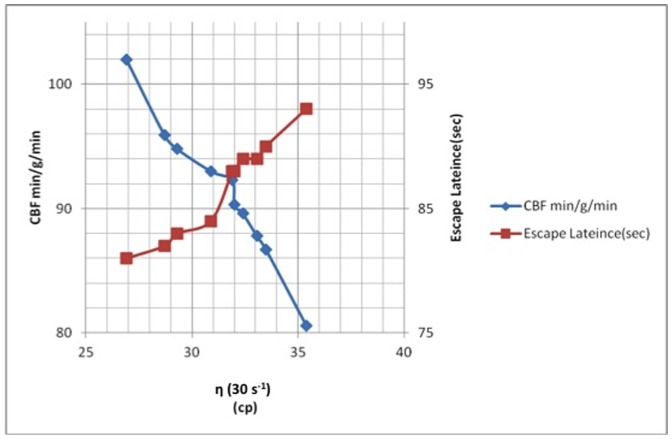
The relationships among ηWB, CBF, and escape latency. The results show that rats with higher whole blood viscosity expressed lower rCBF and longer escape latency.

Tables 3 and 4 show a comparison of complete blood count (CBC) parameters for the rats. Regardless of whether the blood sample was taken from the jugular or the abdominal vein, no significant differences between the control group and the (+)control group were found for any of the CBC parameters. However, Hgb and Hct from the jugular blood were significantly higher than that of the abdominal vein blood in both groups. Tables 5 and 6 show a comparison of hemorheological parameters taken from the jugular blood and the abdominal vein blood of the rats. Except for TK and all other hemorheological parameters were significantly different between the control group and the (+)control group. The WBV, plasma viscosity, and RAI in the (+)control group are significantly higher than in the control group, whereas the EI and OTE in the (+)control group were significantly lower than in the control group. In the (+)control group, WBV of the jugular blood was significantly higher than that of the abdominal vein blood at a low shear rate compared with the control group. Furthermore, an alignment analysis of the relationship among rCBF, hemorheological analysis, and cognitive ability showed that rats with higher WBV expressed lower rCBF and longer escape latency (Figure 6).

**Table 3:** Hematological parameters fromthejugular blood of rats in the control and (+)control groups show no significant differences.

Parameters	Control (n = 10)	(+) Control (n = 10)	P Value
WBC (×10^3^/mm^3^)	2.4±0.7	2.2±0.9	N/S
RBC (×10^6^/mm^3^)	8.58±0.29	8.79±0.28	N/S
Hgb (g/dl)	15.7±0.5	15.9±0.5	N/S
Hct (%)	45.1±1.4	45.9±0.7	N/S
MCV	52.5±1.2	52.2±1.5	N/S
MCH	18.3±0.7	18.1±0.4	N/S
MCHC	34.8±1.0	34.6±1.0	N/S
Platelet (×10^3^/µl)	992.4±59.6	966.5±96.5	N/S

Data are presented as mean ± SD.

**Table 4:** Hematological parameters fromthe abdominal vein blood of rats in the control and (+)control groups show no significant differences.

Parameters	Control (n = 10)	(+) Control (n = 10)	P Value
WBC (×10^3^/mm^3^)	1.9±1.2	1.6±1.0	N/S
RBC (×10^6^/mm^3^)	8.12±0.32	8.23±0.26	N/S
Hgb (g/dl)	14.6±0.6	14.9±0.5	N/S
Hct (%)	42.4±1.8	43.7±1.3	N/S
MCV	52.2±1.4	53.1±1.1	N/S
MCH	18.0±0.3	18.1±0.3	N/S
MCHC	34.4±0.6	34.2±0.8	N/S
Platelet(×10^3^/µl)	1022.7±65.4	916.4±222.3	N/S

Data are presented as mean ± SD.

**Table 5:** Hematological parameters fromthejugular blood of rats in the control and (+)control groups.

Parameters	Control (n = 10)	(+) Control (n = 10)	P Value
ηWB (cp)			
r = 120 S^−^	7.37±0.87	9.52±0.42	<0.01
r = 70 S^−^	8.63±0.90	10.91±0.58	<0.01
r = 30 S^−^	12.49±0.89	15.57±0.68	<0.01
ηP	1.85±0.07	2.20±0.20	<0.01
RAI	4.85±0.56	6.12±0.73	<0.01
EI	13.10±1.07	15.59±1.34	<0.01
TK	0.94±0.07	0.96±0.09	N/S
OTE	0.062±0.007	0.048±0.002	<0.01

ηWB: viscosity of whole blood, cp = mpa•s; ηP: viscosity of plasma; RAI: RBC aggregation index: EI: erythrocyte electrophoresis indexes; TK: internal viscosity of erythrocyte, OTE: oxygen transport efficiency or oxygen delivery index of whole blood = Hct/η WB.

Data are presented as mean ± SD.

**Table 6:** Hematological parameters fromthe abdominal vein blood of rats in the control and (+)control groups.

Parameters	Control (n = 10)	(+) Control (n = 10)	P Value
ηWB(cp)			
r = 120 S^−^	6.34±0.68	8.40±0.67	<0.01
r = 70 S^−^	7.50±0.94	9.71±0.84	<0.01
r = 30 S^−^	11.22±0.88	13.71±0.89	<0.01
ηP	1.79±0.14	2.08±0.12	<0.01
RAI	4.68±0.55	5.95±0.79	<0.01
EI	12.91±0.81	15.39±0.84	<0.01
TK	0.94±0.07	0.95±0.05	N/S
OTE	0.067±0.008	0.051±0.004	<0.01

Data are presented as mean ± SD.

### Aβ Deposits in the Neurons and Vessels of the Hippocampus and Cortex

**Figure 7:**
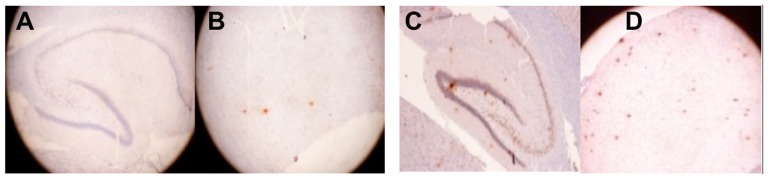
The IHC stain results of the 2 groups. The Aβ40 plaque accumulation in the hippocampus and cortex was monitored using a microscopic examination (100×) and is indicated by the red dye. In the (+)control group (**C** and **D**), significantly more Aβ-containing plaques accumulated in the hippocampus and cortex than in the control group (**A** and **B**).

**Figure 8:**
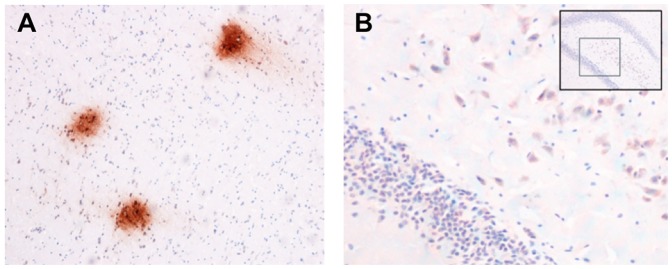
Advanced IHC stain result of the rats with AlCl_3_-induced AD. (**A**)The IHC stain shows Aβ deposits in diffuse plaques. (**B**) The IHC stain shows Aβs (red) accumulating around neurons.

Significantly more Aβ-containing plaques accumulated in the hippocampus and cortex of the rats with AlCl_3_-induced AD(Figure 7) based on IHC staining and visualizing Aβ40, which were conducted using a monoclonal Aβ40 antibody kit. The Aβ deposits in diffuse plaques (Figure 8A) were frequently observed accumulating around neurons (Figure 8B).

## Discussion

### Changes in Cerebral Neuropathology and Cognitive Ability in Rats with AlCl_3_-induced AD

Enigmatic peptide Aβ deposition was proven to be a central event in the neuropathology of AD [1]. The Aβ formed insoluble fibrillar aggregates that accumulated in association with cerebral parenchyma and vasculature cells [30]. The Al is implicated in the etiology of neurodegenerative diseases including AD [31]. Evidence proved that Al significantly contributes to AD [32].Our studies on the changes in cerebral neuropathology indicated that rats in the AlCl_3_-induced AD group exhibited significantly more Aβ-containing plaques in the hippocampus and cortex compared to the control group. The Al replaces calcium and magnesium in the brain. It then combines with the glutamic acid and arginine of amino acid chains to form a stable compound of glutamic acid salt and an arginine salt, which are finally deposited in the brain. The Al also combines with transferrins in the blood and is deposited in the brain cortex, hippocampus, and amygdala. These regions are rich in glutamate neurons; therefore, an abnormal protein phosphorylation reaction occurs, ultimately resulting in the formation of the amyloid precursor protein and yielding Aβsenile plaques and neurofibrillary tangles [33], [34]. The Al can block the induction of long-term potentiation (LTP) in the hippocampal CA3 area of rats [35]. This study evaluated the memory and learning abilities of rats by using the Morris water maze test, which showed that the control group spent a significantly shorter time searching for the target platform and traveled shorter distances to reach the target platform compared to the (+)control group (*P*<0.05) during memory recall tasks. Moreover, the control group frequently spent a longer time lingering in the target quadrant during probe trials compared to the (+)control group. The results clearly indicate that the control rats have superior memory and learning ability, which enables them to intentionally find the escape platform in the water maze test. In contrast, rats with AlCl_3_-induced AD swam along the wall of the pool without attention to direction, indicating that the (+)control rats were unable to preserve and retrieve neural messages. The AlCl_3_-impaired manifestation of the rats in the Morris water maze test [24] causes significant cognitive impairment [20]. The brains of AD patients have higher mean Al concentrations in the hippocampus [36].

### AlCl_3_-induced Oxidative Stress and Aβ

The Aβ plaque accumulation results in cerebral amyloid angiopathy and oxidative stress in the brain and exacerbates the impairment of learning and memory abilities. In turn, oxidative stress triggers Aβ accumulation in an aging brain or a brain with sporadic AD. Our study shows that rats with AlCl_3_-induced AD exhibit greater Aβ-containing plaques accumulating in the hippocampus, cortex, and cerebral vessels, increased MDA content in the erythrocytes of jugular blood and the supernatant of the cerebral cortex and hippocampus, and reduced glutathione peroxidase, superoxide dismutase, and catalase in the supernatant of the cerebral cortex and hippocampus compared with control rats. A previous study showed that Aβ40 infusion increases acetylcholinesterase activity, reactive oxygen species, and lipid peroxidation, and reduces total antioxidant status and superoxide dismutase activity in the brain [37], possibly because the accompanying Aβ plaque formation activates local as well as microglial and astrocytic inflammatory responses, thereby producing oxidative stress.

In addition, aluminum is extremely reactive with carbon and oxygen,and compromises the integrity of the BBB and increasesits permeability [38]–[40]. Chronic exposure to aluminum through drinking water mayAβ-independently and selectively increase inflammatory processesin the central nervous system [41]. Therefore, several reported results may be Aβ-independent.

### β-amyloids and Hemorheological Changes

This study shows that rats with AlCl_3_-induced AD exhibited greater accumulation of Aβ-containing plaques in the hippocampus, cortex, and cerebral vessels, which increases vascular resistance [42], alters hemorheological behavior, and contributes to endothelial dysfunction and vasculopathy [43], [44]. In addition, Aβ-containing plaque accumulation induces RBC aggregation and causes changes in the shear stress of the vessel wall, thereby increasing WBV. Dense Aβ plaques appear to initially develop along blood vessels [45], [46]. Mohanty showed that the red blood cell (RBC) morphology in AD subjects is altered, and these alterations in the RBC membrane architecture are possibly due to RBC-Aβinteractions and changes in the expression of membrane proteins [47]. The Aβ induces oxidative injury to RBCs by binding to them, causing RBC phospholipid peroxidation. The Aβ fibrils can induce erythrocyte adhesion to endothelial cells [48]. The adherence of RBCs to the endothelium reduces the CBF, impairing oxygen delivery to the brain, thus contributing to cerebral hypoxia.

### AlCl_3_-induced Oxidative stress and Hemorheological Changes

AD is associated with abnormal changes of the erythrocyte membrane [47]. Exposure to oxidative stress can dramatically change the function of the erythrocyte membrane, reducing erythrocyte deformability, increasing erythrocyte aggregation, and elevating viscosity [49]–[51]. This study showed decreased erythrocyte EI in rats with AlCl_3_-induced AD caused by erythrocyte membrane abnormality because no significant differences in the TK value existed between the 2 groups. The reduced erythrocyte deformability could result in elevating WBV at a high shear rate and impair the cerebral OTE. Moreover, rats with AlCl_3_-induced AD exhibited a high concentration of plasma fibrinogen, which markedly increased erythrocyte aggregation and plasma viscosity as well as increased WBV at a low shear rate.

Fibrinogen is a large glycoprotein that is synthesized from the liver, circulates in the blood at micromolar concentrations, and can be converted into insoluble fibrin, which is essential for blood coagulation. Previous studies have shown several lines of evidence for the role of fibrinogen in the pathology of AD; Aβ plaques ties fibrinogen leading to circulatory deficiencies. In vitro and in vivo experiments have shown that fibrin clots formed in the presence of Aβ are structurally abnormal and resistant to degradation. Fibrinogen was observed in blood vessels positive for amyloids in mouse and human AD samples [52] and AD patients. High concentrations of fibrinogen have been associated with an increased risk for AD patients and a surged risk for dementia transformation in patients with mild cognitive impairment (MCI); depleting fibrinogen reduced cerebral amyloid angiopathy and cognitive impairment in AD mice [52]. Therefore, we suggest a pathogenic role for fibrinogen in which high fibrinogen concentrations accelerate erythrocyte aggregation, increase plasma viscosity, promote adhesion to Aβ plaques, and impede the clearance of Aβ, causing a change of fibrin clotting that impairs cerebral blood flow and elevates inflammation, thus contributing to cognitive decline in rats with AlCl_3_-induced AD.

### Hemorheological Changes and Regional Cerebral Blood Flow

Blood flow is not affected by Aβs in the heart and kidneys. The Aβ vasoactivity is specific to the cerebral vasculature in both rats and humans, in which Aβ40 exhibited similar potency on cerebral vessels [42]. The clinical correlation between hypoxia and the increased incidence of AD has been well described. Cerebral hypoxia triggers hypometabolic, degenerative, and cognitive alterations in the brain and contributes to the pathology of AD [53]. In addition to the mentioned hemorheology results, we used ASL-MRI and MRA techniques to initially measure the rCBF of rats. Rats with AlCl_3_-induced AD showed a significantly reduced rCBF in the hippocampus and cortex compared with that of the control group (*P*<0.01), and the rCBF of the hippocampus was lower than that of the cortex. We also found that most vessels of the hippocampus and cortex could not be excited using radio frequencies (RFs), which is possibly due to the direct and specific constrictive effect of Aβs on cerebral vessels and may contribute to cerebral hypoperfusion [42]. Two consistent findings in the pathologic ultrastructure of cerebral capillaries regarding AD that influence the laws of fluid dynamics are basement membrane thickening [54] and intramural amyloid deposits (dysphoric angiopathy) [55], [56]. In addition, we found anapparent inverse relationship between WBV and rCBF in rats with AlCl_3_-induced AD.

### Changes in Neurochemicals

MRS plays a crucial role in the early diagnosis of AD. Similar to using structural MRI, using MRS for rat models requires high-resolution scanning to reduce the signal-to-noise ratio (SNR). The results strongly match those of humans and can be used for longitudinal studies that are considerably shorter than those for human cohorts [57].In this study, we used MRS to preliminarily measure the content of NAA. Rats with AlCl_3_-induced AD showed significantly lower levels of NAA. This result isconsistent with those of previous studies [25], [58]. NAA is primarily a marker of neuronal viability and integrity. A previous study showed that APP(Swe)/PS1(dE9) transgenic mice had a significantly decreased hippocampal NAA total creatine (tCr) levels, which are associated with the degeneration and intracellular deposition of Aβ aggregates in hippocampal CA3 pyramidal neurons [59]. In addition to the results listed in Table 3, this shows that the concentration of AChE-RBC in rats with AlCl_3_-induced AD is significantly higher than that of control rats, indicating that neurotransmission functions might be severely affected in rats with AlCl_3_-induced AD. Acetylcholinesterase (AChE) is a senile plaque component that promotes amyloid fibril assembly and the formation of highly toxic Aβ-AChE. The Aβ-AChE complexes induce a greater neurotoxic effect than that induced by the Aβ peptide alone, as shown both in vitro (hippocampal neurons) and in vivo (the Aβ peptide was injected into the dorsal hippocampus of rats) [60], [61]. TheAChE-RBC derived from nerve tissue, muscle, and erythrocyte membrane resembles the AChE of the nervous system, which has a high decomposing ability against acetylcholine and enables nerve cell repetitive depolarization.

### Neurovascular Uncoupling and Impaired Cognitive Abilities

This study showed that rats with AlCl_3_-induced AD exhibited significantly reduced neuron activity and rCBF. Adequate rCBF to neurons protects the tight dynamic relationship between the cellular elements of the neurovascular unit and homeostasis of the cerebral microenvironment (neurovascular coupling), which enhances the cognitive abilities of the brain. Zlokovic indicated that microvascular abnormalities lead to a faulty BBB clearance of Aβs through the deregulated low-density lipoprotein-related protein 1 and receptor for advanced glycation end-product (RAGE)-mediated transport. These impaired clearances of Aβs and glycation end-products result in aberrant angiogenesis, remodeling of the cerebral microvasculature, and eventual arterial dysfunction, which in turn causes neurovascular uncoupling [62]. The progressive neurological impairment in AD is associated with a significant increase in Aβsenile plaque and neurofibrillary tangle counts [63].

**Figure 9:**
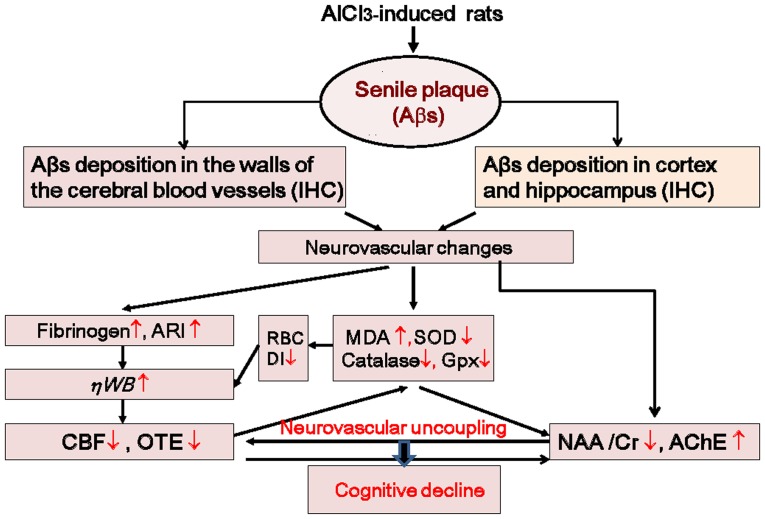
All pathway relationships among Aβs, neurovascular changes, and cognitive decline in rats with AlCl_3_-induced AD. An increase of Aβ plaques induces oxidative stress and, thus, increases the WBV and reduces the CBF and OTE. Moreover, rats with AlCl_3_-induced AD exhibit a high concentration of plasma fibrinogen, which increases erythrocyte aggregation, plasma viscosity, and WBV. In contrast, Aβ plaques increase and deposit in the neurons of the hippocampus and cortex, increasing the activity of AChE and reducing the levels of NAA and Cr. The sustained neurovascular uncoupling ultimately results in cognitive decline.

These data suggest that a rational mechanism exists for both the hemodynamic and neuropathological changes in rats with AlCl_3_-induced AD. When Aβ plaques increase, oxidative injury to the erythrocyte membrane causes a reduction in erythrocyte deformability, an elevation of plasma fibrinogen, and erythrocyte aggregation. This leads to the adherence of RBCs to the endothelium and, thus, increases the WBV, thereby reducing the CBF and OTE. In addition, the obstructions caused by Aβ plaques found on the affected vessel walls might directly interfere with brain microcirculation [8]. In contrast, Aβ-containing plaques deposited in the hippocampus and cortex lead to the formation of a highly neurotoxic Aβ-AChE complex, reducing the NAA level and neural activation. Therefore, the sustained neurovascular uncoupling hampers the cerebral microenvironment and ultimately results in cognitive decline (Figure 9).

## Conclusion

These results underscore the importance of hemorheology and reinforce the specific association between hemodynamic and neuropathological changes in rats with AlCl_3_-induced AD. The results revealed that the insufficient rCBF in rats with AlCl_3_-induced AD was caused by increasing the Aβ plaque accumulation and altering abnormal hemorheological parameters such as erythrocyte deformability, erythrocyte aggregation, OTE, and fibrinogen concentrations, which subsequently increased the WBV. The Aβ plaques were also found to debilitate the activity of neurons by elevating AChE concentration and reducing the NAA content in rats with AlCl_3_-induced AD. These effects have been confirmed by the behavior tasks, MRI data, and IHC staining of Aβs in the rat brain. This study featured hemorheology, CBF testing, MRS, MRA, and IHC staining to demonstrate the hemodynamic and neuropathological changes in rats with AlCl_3_-induced AD. Hemorheological parameters, such as WBV and fibrinogen, coupled with AChE-RBC may be useful biomarkers for disease progression evaluations and the therapeutic monitoring of AD patients that can be used instead of invasive inspections.
